# Pediatric Thymoma: A Review and Update of the Literature

**DOI:** 10.3390/diagnostics12092205

**Published:** 2022-09-12

**Authors:** Cristiana Rossi, Magda Zanelli, Francesca Sanguedolce, Maurizio Zizzo, Andrea Palicelli, Linda Ricci, Matteo Corsi, Cecilia Caprera, Camilla Cresta, Francesco Sollitto, Giuseppe Broggi, Rosario Caltabiano, Alberto Cavazza, Filippo Lococo, Domenico Loizzi, Stefano Ascani

**Affiliations:** 1 Pathology Unit, Azienda Unità Sanitaria Locale ASL5, 19124 La Spezia, Italy; 2 Pathology Unit, Azienda Unità Sanitaria Locale—IRCCS di Reggio Emilia, 42123 Reggio Emilia, Italy; 3 Pathology Unit, Azienda Ospedaliero-Universitaria, Ospedali Riuniti di Foggia, 71122 Foggia, Italy; 4 Surgical Oncology Unit, Azienda USL—IRCCS di Reggio Emilia, 42123 Reggio Emilia, Italy; 5 Pathology Unit, Azienda Ospedaliera S. Maria di Terni, University of Perugia, 05100 Terni, Italy; 6 Institute of Thoracic Surgery, University of Foggia, 71122 Foggia, Italy; 7 Department of Medical and Surgical Sciences and Advanced Technologies “G.F. Ingrassia” Anatomic Pathology, University of Catania, 95123 Catania, Italy; 8 Thoracic Surgery, Fondazione Policlinico Universitario Agostino Gemelli IRCCS, Università Cattolica del Sacro Cuore Roma, 00168 Roma, Italy

**Keywords:** thymoma, pediatric thymoma, mediastinal tumor

## Abstract

Pediatric thymomas are extremely rare and slow-growing malignant tumors. The recent publication of the first Union for International Cancer Control (UICC)/American Joint Committee on Cancer (AJCC) Tumor–Node–Metastasis (TNM) stage classification and updated treatment guidelines for thymomas has prompted us to perform a review of the literature on pediatric thymomas. A search of English-language articles in the PubMed, Cochrane, Web of Science, and Embase databases was conducted. Additional articles were identified through reference lists of retrieved publications. Thirty-two articles involving 82 pediatric thymomas were included. Males comprised 60% of patients, and 13% manifested myasthenia gravis (MG). Histotype B1 (45%) and stage I (52% Masaoka–Koga and 71% UICC/AJCC TNM) were the most frequent. Of note is the possibility that the lack of cases with mixed histologies in the reviewed publications might be related to a sampling issue, as it is well known that the more sections are available for review, the more likely it is that the majority of these neoplasms will show mixed histologies. Both staging systems showed a gradual increase in the percentage of cases, with more advanced stages of disease moving from type A to B3 thymomas. Complete surgical resection (R0) was the main therapeutic approach in Masaoka–Koga stage I (89%) and UICC/AJCC TNM stage I (70%) thymomas. Advanced stages of disease and incomplete surgical resection were most often associated with recurrence and death. An association between stage and outcome, and completeness of resection and outcome, was found. Interestingly, though an association between histotype and staging was found, this does not take into account the possibility of mixed histologies which would reduce the clinical impact of histologic subtyping over staging.

## 1. Introduction

Thymomas are malignant thymic epithelial tumors (TETs) arising primarily in the prevascular (anterior) mediastinum [1,2]. Thymomas are rare neoplasms, but they are the most common TETs. Although thymomas can develop at any age, they are more common in adults, among whom they are the most frequent mediastinal lesion, while they are extremely rare and a less common cause of mediastinal masses in the pediatric population [2,3,4,5,6,7,8]. Thymomas are slow-growing tumors, asymptomatic in nearly one-third of patients in whom the neoplasm is discovered incidentally during imaging studies. A further one-third of patients, approximately, present with signs and symptoms of compression related to a mediastinal mass, many of which are non-specific, overlapping with other pathologies. As a consequence, the diagnosis of pediatric thymomas may be very challenging. Multiple autoimmune and paraneoplastic (AI/PN) disorders have been associated with thymomas [9], the most common being myasthenia gravis (MG), whose association with thymomas is more frequently reported in adults (38%) [10] than in the pediatric population (15%) [11].

Controversy had long surrounded the histopathological classification of TETs [12] until 1999, when the World Health Organization (WHO) proposed a classification schema which represents a sort of “translator” between the different schemas of Bernatz and Muller-Hermelink [13].

The subsequent editions of the WHO classification, including the most recent, have largely maintained the original approach, which identifies five different histotypes (A, AB, B1, B2, and B3) [2]. The first Union for International Cancer Control (UICC)/American Joint Committee on Cancer (AJCC) Tumor–Node–Metastasis (TNM) stage classification for TETs was published in 2017 [14,15] after a forty-year debate and fifteen different staging systems were developed by various authors [16]. Among them, the Masaoka staging system [17] and its revision, the Masaoka–Koga staging system [18], have been the most widely used and accepted for more than thirty-five years [19,20].

Surgery is considered the mainstay of treatment for all resectable thymomas. Radical thymectomy is recommended for most resectable tumors, while thymomectomy should be an option in selected cases. Lymphadenectomy is routinely recommended according to the guidance of the International Thymic Malignancy Interest Group (ITMIG)/International Association for the Study of Lung Cancer (IASLC). Although the median sternotomy is the standard approach, minimally invasive procedures (transcervical, extended transcervical, video-assisted thoracoscopy, and robotic surgery) may be performed in specialized centers by expert thoracic surgeons. Adjuvant radiotherapy may be considered or recommended on the basis of the stage and completeness of resection, while adjuvant chemotherapy is not recommended for resectable thymomas. Advanced thymomas should be managed with primary chemotherapy if complete surgical resection (R0) is deemed achievable, or with definitive radio- and/or chemotherapy [21,22].

Our aim is to provide a comprehensive clinicopathological review of all pediatric thymomas reported over the last thirty-five years in the English literature. 

## 2. Materials and Methods

A search of English-language articles in the PubMed, Cochrane, Web of Science, and Embase databases from 1 January 1985 to 31 December 2020 was conducted using the following subject headings and keywords: “thymoma”, “thymic epithelial tumors”, “pediatric”, “childhood”, “child”, “children”, “juvenile”, and “adolescent”. The selection criteria included: diagnosis of thymoma and patients under the age of eighteen. Cases with a diagnosis of thymic carcinoma, thymic neuroendocrine tumors, and combined thymic carcinomas were excluded. Additional articles were identified and included through the reference lists of retrieved case reports and series. We created a retrospective database which comprised age, sex, clinical presentation, thymoma subtype, Masaoka–Koga stage, UICC/AJCC TNM classification and stage, local invasion into adjacent structures, regional lymph node involvement, distant metastasis, treatment, outcome, and follow-up. 

We acknowledge, however, that the retrospective database has some limitations: probable redundant reports, possible incompleteness of available data, different follow-up periods, diverse interpretations of the same data by different authors, and changeable classifications and definitions over time. Specifically, we recorded the WHO histotype [2] when reported by the referenced article, whereas we retained the original description of the thymoma type when a WHO histotype was not assigned. Regarding staging, none of the articles unequivocally reported the Masaoka–Koga [18] or the UICC/AJCC TNM stage [14,15]. When the original data allowed it, we assigned both the Masaoka–Koga stage, in light of clarifications provided by the ITMIG [19], and the UICC/AJCC TNM stage. In cases of interpretative doubt concerning two or more Masaoka–Koga and UICC/AJCC TNM stages, we reported the various possible alternatives, whereas, when the original articles provided confusing and contradictory data, or no data regarding staging, we did not assign any Masaoka–Koga or UICC/AJCC TNM stage.

## 3. Results

Thirty-two articles, twenty-three case reports [23,24,25,26,27,28,29,30,31,32,33,34,35,36,37,38,39,40,41,42,43,44,45], seven small retrospective case series [46,47,48,49,50,51,52], and two reviews of the literature [11,53] were identified, for a total of eighty-two pediatric thymomas (Table S1). Patients ranged in age from 0.9 to 17 years, with a mean age of 10.5 years. Of the 77 patients whose gender was available, 46 (60%) were male and 31 (40%) were female. Clinically, 16 of the 82 patients (20%) had AI/PN associated disorders: 11 (13%) manifested MG, 2 (2%) aplastic anemia, 2 (2%) hypogammaglobulinemia, and 1 (1%) systemic lupus erythematosus.

Of the 44 cases with WHO histopathological classification, 3 (7%) were WHO type A thymomas, 6 (14%) type AB, 20 (45%) type B1, 12 (27%) type B2, and 3 (7%) type B3. Type B1 thymoma was the most frequent. No cases with mixed histological types were found in the revised articles; neither did we find any mention of the number of tumor sections examined. The WHO histopathological classification, reported in 7 of 11 patients with MG, was as follows: 3 (3/20 = 15%) type B1, 3 (3/12 = 25%) type B2, and 1 (1/3 = 33%) type B3. 

Of the 67 cases in which the Masaoka–Koga stage could be assigned, 35 (52%) had stage I, 1 (1%) had stage IIa, 8 (12%) had stage IIb, 9 (13%) had stage III, 1 (1%) had stage IVa, 8 (12%) had stage IVb, and 5 (7%) gave rise to interpretive doubts between two or more stages. Of the 66 cases in which the UICC/AJCC TNM stage could be assigned, 47 (71%) were stage I, 1 (2%) was stage II, 4 (6%) were stage IIIa, 2 (3%) were stage IIIb, 1 (2%) was stage IVa, 8 (12%) were stage IVb, and 3 (5%) gave rise to interpretive doubts between two or more stages.

**Figure 1:**
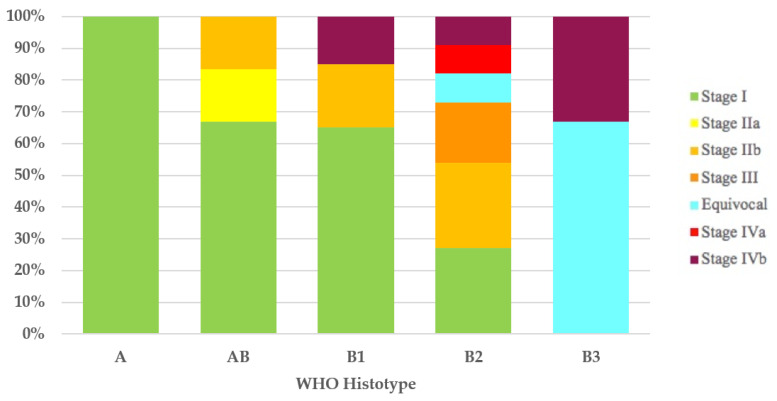
Stage distribution in thymoma histotypes according to the Masaoka–Koga staging system. WHO, World Health Organization.

It was possible to assign the Masaoka–Koga stage to 43 of the 44 thymomas with the WHO histotype. All type A (100%) thymomas and most type AB (4/6 = 67%) and type B1 (13/20 = 65%) thymomas had Masaoka–Koga stage I. In 11 of 12 type B2 thymomas, the Masaoka–Koga stage was available, and these were as follows: 3 cases (27%) were stage I, 3 (27%) were stage IIb, 2 (18%) were stage III, 1 (9%) was equivocal stage (Case 49), 1 (9%) was stage IVa, and 1 (9%) was stage IVb. Of the three type B3 thymomas, two (67%) had equivocal Masaoka–Koga stage (Cases 1 and 30) and the third case had Masaoka–Koga stage IVb (Figure 1).

**Figure 2:**
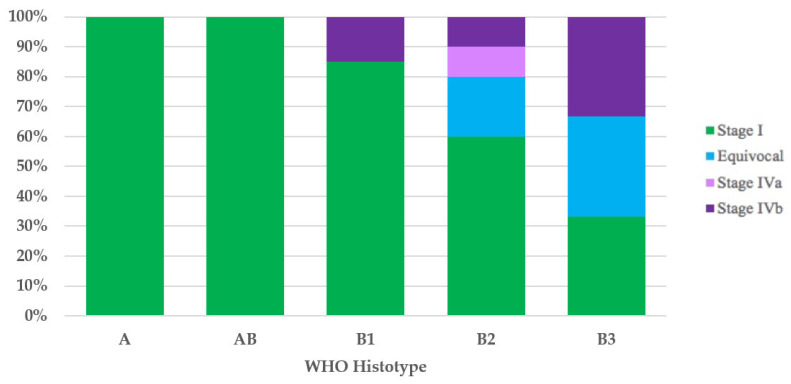
Stage distribution in thymoma histotypes according to the UICC/AJCC TNM stage classification. UICC/AJCC TNM, Union for International Cancer Control/American Joint Committee on Cancer Tumor–Node–Metastasis; WHO, World Health Organization.

It was possible to assign the UICC/AJCC TNM stage to 42 of the 44 thymomas with the WHO histotype. All type A (100%) and type AB (100%) thymomas, and most type B1 (17/20 = 85%) thymomas had UICC/AJCC TNM stage I. Ten of twelve type B2 thymomas had the UICC/AJCC TNM stage available, and these were as follows: six cases (60%) stage I, two (20%) equivocal stage (Cases 49 and 52), one (10%) stage IVa, and one (10%) stage IVb. Of the three type B3 thymomas, the first (33%) had UICC/AJCC TNM stage I, the second (33%) had equivocal UICC/AJCC TNM stage (Case 1), and the third case had UICC/AJCC TNM stage IVb (Figure 2).

**Figure 3:**
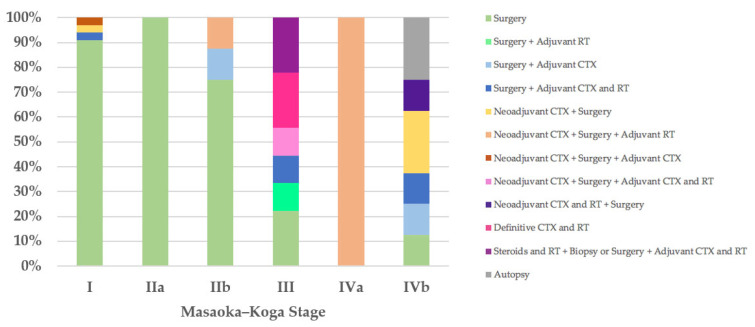
Treatment according to Masaoka–Koga stage. CTX, chemotherapy; RT, radiotherapy.

Regarding the therapeutic approach according to the Masaoka–Koga staging system, of the 35 stage I patients, 32 (91%) were treated exclusively with surgery, in 31 cases (89%) with R0 surgery and only in 1 case with microscopically incomplete resection (R1) followed by further R0 surgery (Case 55). Three stage I patients received neoadjuvant and/or adjuvant therapies and R0 surgery (Cases 13, 22, and 71). The only patient (100%) with stage IIa was treated with R1 surgery (Case 21). Of the eight stage IIb patients, six (75%) underwent surgery, R0 in five cases (63%) and R1 in one case followed by further R0 surgical resection (Case 18), and two patients received neoadjuvant and/or adjuvant therapies in addition to R0 or R1 surgery (Cases 8 and 9). Two of the nine patients with Masaoka–Koga stage III were treated exclusively with surgery with macroscopically incomplete resection (R2) (Cases 52 and 76); two patients were treated with R2 surgery followed by adjuvant therapies (Cases 62 and 81); one patient with unresectable thymoma underwent biopsy and then neoadjuvant chemotherapy followed by R1 surgery and adjuvant chemoradiotherapy (Case 26); two patients (22%) with unresectable thymoma underwent biopsy and then definitive chemoradiotherapy (Cases 63 and 79); and two patients were treated with steroids and radiotherapy, were then deemed unresectable, and underwent biopsy or R2 surgery followed by adjuvant chemoradiotherapy (Cases 77 and 78). The only patient (100%) in Masaoka–Koga stage IVa, with unresectable thymoma, underwent biopsy and then neoadjuvant chemotherapy followed by R2 surgery and adjuvant radiotherapy (Case 56). Of the eight stage IVb patients, one (13%) was treated exclusively with surgery (Case 32); two patients (25%) with unresectable thymoma underwent biopsy and then neoadjuvant chemotherapy followed by R1 or R2 surgery (Cases 20 and 51); one patient (13%) was treated with neoadjuvant chemoradiotherapy followed by surgery (Case 42); two patients underwent R2 surgery followed by adjuvant therapies (Cases 74 and 75); and in two cases (25%) the diagnosis was made on autopsy (Figure 3).

**Figure 4:**
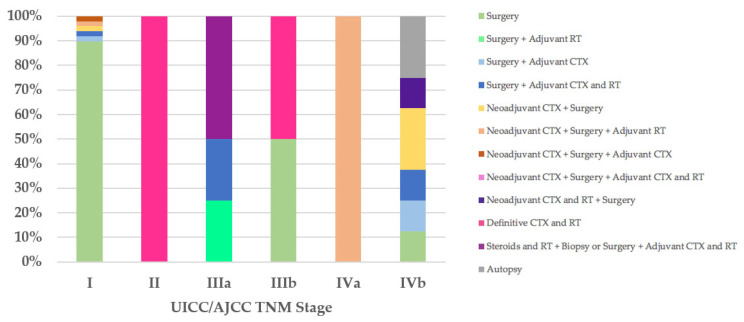
Treatment according to UICC/AJCC TNM stage. CTX, chemotherapy; RT, radiotherapy; UICC/AJCC TNM, Union for International Cancer Control/American Joint Committee on Cancer Tumor–Node–Metastasis.

Regarding the therapeutic approach according to the UICC/AJCC TNM stage classification, of the 47 stage I patients, all T1aN0M0, 42 (89%) were treated exclusively with surgery: R0 in 39 cases, 33 (70%) of which did not have macroscopic infiltration into the thymic capsule or surrounding fatty tissue; R1 in three cases (Cases 18, 21, and 55), two of which went for further R0 surgery. Five stage I patients received neoadjuvant and/or adjuvant therapies and R0 or R1 surgery (Cases 8, 9, 13, 22, and 71). The only patient (100%) in UICC/AJCC TNM stage II, with unresectable thymoma, underwent biopsy and then definitive chemoradiotherapy (Case 79). Of the four stage IIIa patients, two (50%) were treated with steroids and radiotherapy, then deemed unresectable and underwent biopsy or R2 surgery followed by adjuvant chemoradiotherapy (Cases 77 and 78), while the other two patients underwent R2 surgery followed by adjuvant therapies (Cases 62 and 81). One of the two patients (50%) with stage IIIb was treated exclusively with R2 surgery (Case 76), while the other (50%), with unresectable thymoma, underwent biopsy and then definitive chemoradiotherapy (Case 63). The therapeutic approach according to the UICC/AJCC TNM IVa and IVb stages coincided with that observed in patients with Masaoka–Koga stages IVa and IVb (Figure 4).

Outcome and follow-up data according to stage and therapy, with particular reference to the completeness of resection, were available for 74 of the 82 patients and are reported in Table 1 and Table 2, respectively.

## 4. Discussion

Thymoma is exceedingly rare in patients under the age of eighteen. 

From 1985 to 2020, we found 82 cases of pediatric thymomas in the English literature. Two previous reviews of the literature included thirty-two [53] and forty-eight [11] cases of pediatric thymomas, respectively. In a large series of more than 1400 cases of thymomas reported in 2018, pediatric thymomas have been estimated to make up around 1% [54]. 

In adults, no gender predilection is reported [10], as in the review of pediatric thymomas by Liang et al. [53]. However, our data highlighted a slightly higher incidence in males (60%), confirming the finding already observed by Fonseca et al. [11]. Our review of the literature showed an incidence of MG in pediatric patients with thymoma of 13%, which is in line with the incidence reported by the two previous literature reviews [11,53] and confirms the lower incidence of MG in the pediatric population compared with adults, in which the incidence of MG is 38% [10]. Furthermore, according to our data, MG is the most common thymoma-associated AI/PN disorder in the pediatric population, as it is in adults [10]. 

Even considering the limit that the WHO histopathological classification was available in only slightly more than half of the cases in our review, B1 thymoma appeared as the most frequent type (45%), while in the adult population the most common type is B2 [10].

However, it is worth emphasizing that no cases with mixed histological types appear to have been encountered in our review of pediatric thymomas. As stated by Moran et al., sampling is an important issue in the proper subtyping of these neoplasms [55]. As we did not find any mention to the number of sections examined, it is likely that the lack of cases with mixed histologies might be strictly related to the fact that a limited number of sections was evaluated. It is well known that the more sections that are available for review, the more likely it is that the majority of these neoplasms will show mixed histologies, which reduces the clinical impact of histologic subtyping over staging [55].

In adults, MG is more frequent in types B1–3 than in type A and AB thymomas [56]. Taking into consideration the limit that the histological type was available in only seven of the eleven patients with MG, our data showed that MG was associated only with type B thymomas.

In 2017, the UICC/AJCC TNM stage classification was introduced [14,15], along with the staging, the nodal map, and the indications for removal and/or sampling of lymph nodes, proposed by the ITMIG/IASLC [10,20,57,58,59,60,61].

It is worth mentioning that the TNM staging system borrowed the majority of the information it used from other well-formulated staging systems which had been tested on large series of cases over the years [62,63].

As highlighted by a recent survey from the IASLC Staging and Prognostic Factors Committee–Thymic Domain (SPFC–TD) [64] analyzing the impact of the UICC/AJCC TNM stage classification in clinical practice, even though TNM classification has received positive recognition by the scientific community, the Masaoka–Koga staging system remains the most used staging system. In addition, although compliance with the nodal map may be considered acceptable, only 48% of those responding stated that they routinely employ the nodal map in clinical practice. Therefore, it is not surprising that the first three cases (Cases 1–3) in our literature review, published after the introduction of the UICC/AJCC TNM stage classification, do not use such staging or the nodal map. The application of the Masaoka–Koga [18] and UICC/AJCC TNM stage classifications [14,15], wherever possible, has enabled us to show how, according to both staging systems, stage I is the most frequent. Furthermore, with the UICC/AJCC TNM stage classification [14,15], compared with the Masaoka–Koga staging system [18], we have observed an increase in stage I thymomas (71% versus 52%, respectively) and the replacement of the Masaoka–Koga stage III with the UICC/AJCC TNM stage IVb as the second most frequent stage. This was an expected finding considering that UICC/AJCC TNM stage I incorporates Masaoka–Koga stages I, IIa, IIb, and a part of stage III, and that Masaoka–Koga stage III is subdivided into UICC/AJCC TNM stages I, II, IIIA, and IIIB [21,65]. The evaluation of the distribution of the stages, by both Masaoka–Koga and UICC/AJCC TNM, in the five thymoma histotypes, has revealed a gradual decrease in the percentage of stage I cases and a progressive increase in the percentage of cases with more advanced stages of disease, while proceeding from type A/AB thymomas to type B1, B2, and especially B3 thymomas. Stages III and IV could be found only in type B thymomas. What we have observed is in contrast with the absence of type A thymomas in stages I and II, as described by Fonseca et al. [11] and as is consistent with the strong association between histotype and stage reported in the adult population [56]. 

Due to the extreme rarity of thymomas in pediatric patients, no specific guidelines have been established for the management of TETs in the pediatric population, and therefore it is reasonable to refer to the position paper by the Italian collaborative group for ThYmic MalignanciEs (TYME) [66], the European Society for Medical Oncology (ESMO) clinical practice guidelines for TETs [21], and the National Comprehensive Cancer Network (NCCN) clinical practice guidelines for thymomas and thymic carcinomas [22]. Although these guidelines differ in part from each other, all agree that surgery is the mainstay of therapy if R0 resection is achievable. Radical thymectomy is recommended for all resectable thymomas, as in the case of Masaoka–Koga stage I and II thymomas and some stage III thymomas; after R0 resection of Masaoka–Koga stage I thymomas, adjuvant treatments are not recommended, whereas after R1 resection of Masaoka–Koga stage I thymomas, adjuvant radiotherapy is recommended. Finally, adjuvant chemotherapy is not recommended for R0 and R1 resected thymomas. Furthermore, regarding locally advanced thymomas, there is unanimous agreement that the standard of care in these cases is primary/induction chemotherapy, preferably with cisplatin-based combination regimens, followed by a reassessment of the patient with imaging, and, if complete resection is deemed feasible, surgery should be performed followed by adjuvant radiotherapy. Finally, the current guidelines agree in recommending that recurrences be managed with the same approach as newly diagnosed thymomas [21,22,66]. As we have seen, the principles of treatment for TETs are predominantly based on the Masaoka–Koga staging system, with the single exception being, to the best of our knowledge, the Italian position paper [66], which was the first to propose specific recommendations based on the UICC/AJCC TNM stage classification. With both staging systems, stage I was the most frequent, and therefore it is not surprising that the main therapeutic approach in our literature review was R0 surgery. Specifically, stage I is the stage in which concordance with the therapeutic approach recommended by the guidelines was most often observed; indeed, 89% of Masaoka–Koga stage I patients and 70% of UICC/AJCC TNM stage I patients were treated exclusively with R0 surgery in accordance with the TYME [66], ESMO [21], and NCCN guidelines [22], and the TYME guidelines [66], respectively. Furthermore, concordance with the therapeutic approach recommended by the TYME [66], ESMO [21], and NCCN guidelines [22] was observed in 63% of Masaoka–Koga stage IIb patients, who were treated exclusively with R0 surgery, and in 22% of Masaoka–Koga stage III thymomas, which were deemed unresectable and therefore underwent biopsy and then definitive chemoradiotherapy. Agreement with the TYME guidelines [66] based on the UICC/AJCC TNM stage classification, in addition to stage I, was observed in one of two patients (50%) with unresectable stage IIIb thymoma undergoing biopsy and then definitive chemoradiotherapy. Finally, the only stage IVa patient with both staging systems was treated for the primary tumor and for the two recurrences, with adherence to guidelines based both on the Masaoka–Koga and UICC/AJCC TNM stage classifications [21,22,66].

Stage and completeness of resection are the most important and consistent independent prognostic factors in terms of recurrence, disease-free survival, and overall survival [56,67,68,69]. Our data, although based on a limited number of patients, show a tendency in line with what has been established by the literature. Indeed, regarding stages, of the nine cases with disease recurrence, seven (78%) with Masaoka–Koga stage classification and six (67%) with UICC/AJCC TNM stage classification showed stage III or higher. In addition, the majority of living patients had Masaoka–Koga stage I (46%) and UICC/AJCC TNM stage I (65%), while the majority of deceased patients had stage III or higher according to the Masaoka–Koga (73%) and UICC/AJCC TNM (64%) stage classifications. Regarding the completeness of resection, of the nine patients with disease recurrence, five (56%) were R1 or R2 (R+) and only one (11%) was R0, and the majority of living patients were R0 (63%) whereas the majority of deceased patients were R+ (36%). The only deceased patient in Masaoka–Koga and UICC/AJCC TNM stage I after treatment with R0 surgery did not die as a direct result of thymoma, but rather from a disseminated varicella infection (Case 80). 

## 5. Conclusions

Our review of the literature confirms that thymomas in the pediatric population, compared with that of adults, are much more rare, slightly more frequent in males, and have a lower incidence of MG. In addition, although performing statistical analyses has not been possible, our data show an association between stage and outcome, and completeness of resection and outcome, in line with the findings of the literature for the adult population. We did not find in the reviewed literature any cases of mixed histologies, which might be due to a sampling issue. Therefore, it needs to be highlighted that the association between histotype and staging, which was found in the reviewed cases, does not take into consideration the possible presence of mixed histologies which would diminish the clinical impact of histologic subtyping over staging.

An increasing use of the new UICC/AJCC TNM stage classification and further studies based on multi-institutional databases are necessary to understand whether significant differences exist in terms of prognostic factors and outcomes among adults and the pediatric population which may lead to defining specific therapeutic protocols for pediatric patients with thymoma.

## Figures and Tables

**Table 1:** Outcome by stage.

		Patients with Outcome and Follow-Up (*n* = 74)					
		Alive (*n* = 63)			Died (*n* = 11)		
Staging System	Stage	No Recurrence	Recurrence	Total (%)	No Recurrence	Recurrence	Total (%)
Masaoka–Koga	I	28 *range FU: 1–108 mos*	1 ^a^ *FU: 12 mos*	29 (46)	1 ^e^ *FU: 4 mos*	0	1 (9)
	IIa	1 *FU: 48 mos*	0	1 (2)	0	0	0 (0)
	IIb	8 *range FU: 12–132 mos*	0	8 (13)	0	0	0 (0)
	III	4 *range FU: 2–240 mos*	0	4 (6)	1 ^f^ *FU: 0.8 mos*	4 ^g^ *range FU: 6–30 mos*	5 (45)
	IVa	0	1 ^b^ *FU: 22 mos*	1 (2)	0	0	0 (0)
	IVb	4 *range FU: 6–209 mos*	1 ^c^ *FU: 15 mos*	5 (8)	3 ^h^ *range FU: 0–13 mos*	0	3 (27)
	equivocal	4 *range FU: 0.27–84 mos*	1 ^d^ *FU: 56 mos*	5 (8)	0	0	0 (0)
	none	10 *range FU: 24–357 mos*	0	10 (16)	1 ^i^ *FU: 0.8 mos*	1 ^j^ *FU: 8 mos*	2 (18)
**Total**		59	4	63 (100)	6	5	11 (100)
UICC/AJCC TNM	I	40 *range FU: 1–132 mos*	1 ^a^ *FU: 12 mos*	41 (65)	1 ^e^ *FU: 4 mos*	0	1 (9)
	II	0	0	0 (0)	0	1 ^g^ *FU: 7 mos*	1 (9)
	IIIa	1 *FU: 10 mos*	0	1 (2)	0	3 ^g^ *range FU: 6–30 mos*	3 (27)
	IIIb	1 *FU: 84 mos*	0	1 (2)	1 ^f^ *FU: 0.8 mos*	0	1 (9)
	IVa	0	1 ^b^ *FU: 22 mos*	1 (2)	0	0	0 (0)
	IVb	4 *range FU: 6–209 mos*	1 ^c^ *FU: 15 mos*	5 (8)	3 ^h^ *range FU: 0–13 mos*	0	3 (27)
	equivocal	2 *range FU: 0.27–2 mos*	1 ^d^ *FU: 56 mos*	3 (5)	0	0	0 (0)
	none	11 *range FU: 24–367 mos*	0	11 (17)	1 ^i^ *FU: 0.8 mos*	1 ^j^ *FU: 8 mos*	2 (18)
**Total**		59	4	63 (100)	6	5	11 (100)

FU, Follow-Up; mos, months; UICC/AJCC TNM, Union for International Cancer Control/American Joint Committee on Cancer Tumor–Node–Metastasis. ^a^ Case 50: recurrent thymoma. ^b^ Case 56: recurrent pleural disease 1 month after diagnosis and recurrent pleural e peritoneal disease 22 months after diagnosis. ^c^ Case 75: local recurrence. ^d^ Case 49: recurrent thymoma. ^e^ Case 80: death from disseminated varicella infection in patient with thymoma and hypogammaglobulinemia and lymphopenia. ^f^ Case 76: died of disease. ^g^ Cases 62, 77, 78, and 79: died of disease (distant metastasis). ^h^ Cases 16, 20, and 46 died of disease; Cases 16 and 46 were diagnosed on autopsy. ^i^ Case 43: died of disease. ^j^ Case 39: died of disease (cervical lymph node metastasis).

**Table 2:** Outcome by completeness of resection.

		Patients with Outcome and Follow-Up (*n* = 74)					
		Alive (*n* = 63)			Died (*n* = 11)		
Resection Status	Treatment	No Recurrence	Recurrence	Total (%)	No Recurrence	Recurrence	Total (%)
R0	surgery	36 ^a^ *range FU: 1–132 mos*	1 ^b^ *FU: 12 mos*	40 (63)	1 ^c^ *FU: 4 mos*	0	1 (9)
	surgery + neoadjuvant and/or adjuvant therapies	3 *range FU: 30–60 mos*	0		0	0	
R+	surgery	2 *range FU: 2–48 mos*	0	11 (17)	1 ^d^ *FU: 0.8 mos*	0	4 (36)
	surgery + neoadjuvant and/or adjuvant therapies	6 *range FU: 6–240 mos*	3 ^e^ *range FU: 15–56 mos*		1 ^f^ *FU: 13 mos*	2 ^g^ *range FU: 6–18 mos*	
R_NA_	surgery	8 *range FU: 0.27–367 mos*	0	10 (16)	0	0	1 (9)
	surgery + neoadjuvant and/or adjuvant therapies	2 *range FU: 209–217 mos*	0		0	1 ^h^ *FU: 8 mos*	
none	chemoradiotherapy	2 *range FU: 40–84 mos*	0	2 (3)	0	2 ^i^ *range FU: 7–30 mos*	2 (18)
none	none	0	0	0 (0)	3 *range FU: 0–0.8 mos*	0	3 (27)
**Total**		59	4	63 (100)	6	5	11 (100)

FU, Follow-Up; mos, months; R0, complete resection; R+, R1 (microscopically incomplete resection) or R2 (macroscopically incomplete resection); RNA, resection status not available. ^a^ Thirty-four patients underwent R0 surgery; two patients (Cases 18 and 55) underwent R1 surgery followed by further R0 surgery. ^b^ Case 50: recurrent thymoma. ^c^ Case 80: death from disseminated varicella infection in patient with thymoma and hypogammaglobulinemia and lymphopenia. ^d^ Case 76: died of disease. ^e^ Case 49: recurrent thymoma; Case 56: recurrent pleural disease 1 month after diagnosis and recurrent pleural e peritoneal disease 22 months after diagnosis; and Case 75: local recurrence. ^f^ Case 20: died of disease. ^g^ Cases 62 and 78: died of disease (distant metastasis). ^h^ Case 39: died of disease (cervical lymph node metastasis). ^i^ Cases 77 and 79: died of disease (distant metastasis).

## Data Availability

Individual patient data from the original studies included in the present review are not applicable, and data sharing at this level is not applicable for a review.

## Supplementary Materials

The following supporting information can be downloaded at: https://www.mdpi.com/article/10.3390/diagnostics12092205/s1, Table S1: Summary of cases. References [11,23,24,25,26,27,28,29,30,31,32,33,34,35,36,37,38,39,40,41,42,43,44,45,46,47,48,49,50,51,52,53] are cited in the Supplementary Materials.

## References

[1] Carter B.W., Marom E.M., Detterbeck F.C. (2014). Approaching the patient with an anterior mediastinal mass: A guide for clinicians. J. Thorac. Oncol..

[2] WHO Classification of Tumours Editorial Board, IARC (2021). WHO Classification of Tumours. Thoracic Tumours.

[3] Takeda S., Miyoshi S., Akashi A., Ohta M., Minami M., Okumura M., Masaoka A., Matsuda H. (2003). Clinical spectrum of primary mediastinal tumors: A comparison of adult and pediatric populations at a single Japanese institution. J. Surg. Oncol..

[4] Tansel T., Onursal E., Dayloğlu E., Başaran M., Sungur Z., Qamci E., Yilmazbayhan D., Eker R., Ertuğrul T. (2006). Childhood mediastinal masses in infants and children. Turk. J. Pediatric.

[5] Gun F., Erginel B., Unüvar A., Kebudi R., Salman T., Celik A. (2012). Mediastinal masses in children: Experience with 120 cases. Pediatric Hematol. Oncol..

[6] Liu T., Al-Kzayer L.F.Y., Xie X., Fan H., Sarsam S.N., Nakazawa Y., Chen L. (2017). Mediastinal lesions across the age spectrum: A clinicopathological comparison between pediatric and adult patients. Oncotarget.

[7] Chen C.H., Wu K.H., Chao Y.H., Weng D.F., Chang J.S., Lin C.H. (2019). Clinical manifestation of pediatric mediastinal tumors, a single center experience. Medicine.

[8] Roden A.C., Fang W., Shen Y., Carter B.W., White D.B., Jenkins S.M., Spears G.M., Molina J.R., Klang E., Segni M.D. (2020). Distribution of mediastinal lesions across multi-institutional, international, radiology databases. J. Thorac. Oncol..

[9] Marchevsky A.M., Suster S., Wick M.R., Marchevsky A.M., Wick M.R. (2000). Low-grade and intermediate-grade malignant epithelial tumors of the thymus: Thymomas. Pathology of the Mediastinum.

[10] Huang J., Ahmad U., Antonicelli A., Catlin A.C., Fang W., Gomez D., Loehrer P., Lucchi M., Marom E., Nicholson A. (2014). Development of the international thymic malignancy interest group international database: An unprecedented resource for the study of a rare group of tumors. J. Thorac. Oncol..

[11] Fonseca A.L., Ozgediz D.E., Christison-Lagay E.R., Detterbeck F.C., Caty M.G. (2014). Pediatric thymomas: Report of two cases and comprehensive review of the literature. Pediatric Surg. Int..

[12] Shimosato Y. (1994). Controversies surrounding the subclassification of thymoma. Cancer.

[13] Rosai J., Sobin L.H. (1999). Histological Typing of Tumours of the Thymus.

[14] Asamura H., Brierley J.D., Gospodarowicz M.K., Wittekind C. (2017). Thymic tumours. TNM Classification of Malignant Tumours.

[15] Detterbeck F.C., Marom E.M., Amin M.B. (2017). Thymus. AJCC Cancer Staging Manual.

[16] Filosso P.L., Ruffini E., Lausi P.O., Lucchi M., Oliaro A., Detterbeck F. (2014). Historical perspectives: The evolution of the thymic epithelial tumors staging system. Lung Cancer.

[17] Masaoka A., Monden Y., Nakahara K., Tanioka T. (1981). Follow-up study of thymomas with special reference to their clinical stages. Cancer.

[18] Koga K., Matsuno Y., Noguchi M., Mukai K., Asamura H., Goya T., Shimosato Y. (1994). A review of 79 thymomas: Modification of staging system and reappraisal of conventional division into invasive and non-invasive thymoma. Pathol. Int..

[19] Detterbeck F.C., Nicholson A.G., Kondo K., Van Schil P., Moran C. (2011). The Masaoka-Koga stage classification for thymic malignancies: Clarification and definition of terms. J. Thorac. Oncol..

[20] Detterbeck F.C., Asamura H., Crowley J., Falkson C., Giaccone G., Giroux D., Huang J., Kim J., Kondo K., Lucchi M. (2013). The IASLC/ITMIG thymic malignancies staging project: Development of a stage classification for thymic malignancies. J. Thorac. Oncol..

[21] Girard N., Ruffini E., Marx A., Faivre-Finn C., Peters S., ESMO Guidelines Committee (2015). Thymic epithelial tumours: ESMO Clinical Practice Guidelines for diagnosis, treatment and follow-up. Ann. Oncol..

[22] NCCN National Comprehensive Cancer Network Home Page. https://www.nccn.org/guidelines/guidelines-detail?category=1&id=1469.

[23] Furman W.L., Buckley P.J., Green A.A., Stokes D.C., Chien L.T. (1985). Thymoma and myasthenia gravis in a 4-year-old child. Case report and review of the literature. Cancer.

[24] Shibata K., Koga Y., Onitsuka T., Karashima S., Sawa S., Murayama T., Kohno M. (1986). Primary malignant thymoma in a 6-year-old boy. Jpn. J. Surg..

[25] Aghaji M.A., Uzuegbunam C. (1990). Invasive thymoma and myasthenia gravis in a three-and-a-half-year-old boy: Case report and literature review. Cent. Afr. J. Med..

[26] Watts R.G., Kelly D.R. (1990). Fatal varicella infection in a child associated with thymoma and immunodeficiency (Good’s syndrome). Med. Pediatric Oncol..

[27] Kaplinsky C., Mor C., Cohen I.J., Goshen Y., Yaniv I., Tamary H., Jaber L., Stark B., Stern S., Zaizov R. (1992). Childhood malignant thymoma: Clinical, therapeutic, and immunohistochemical considerations. Pediatric Hematol. Oncol..

[28] Lam W.W., Chan F.L., Lau Y.L., Chau M.T., Mok C.K. (1993). Paediatric thymoma: Unusual occurrence in two siblings. Pediatric Radiol..

[29] Sicherer S.H., Cabana M.D., Perlman E.J., Lederman H.M., Matsakis R.R., Winkelstein J.A. (1998). Thymoma and cellular immune deficiency in an adolescent. Pediatric Allergy Immunol..

[30] Dhall G., Ginsburg H.B., Bodenstein L., Fefferman N.R., Greco M.A., Chang M.W., Gardner S. (2004). Thymoma in children: Report of two cases and review of literature. J. Pediatric Hematol. Oncol..

[31] Trobaugh-Lotrario A.D., Liang X., Janik J.S., Lovell M.A., Odom L.F. (2004). Difficult diagnostic and therapeutic cases: CASE 2. thymoma and tumor lysis syndrome in an adolescent. J. Clin. Oncol..

[32] Rothstein D.H., Voss S.D., Isakoff M., Puder M. (2005). Thymoma in a child: Case report and review of the literature. Pediatric Surg. Int..

[33] Honda S., Morikawa T., Sasaki F., Okada T., Naito S., Itoh T., Kubota K., Todo S. (2007). Cystic thymoma in a child: A rare case and review of the literature. Pediatric Surg. Int..

[34] Coulter D., Gold S. (2007). Thymoma in the offspring of a patient with Isaacs syndrome. J. Pediatric Hematol. Oncol..

[35] Ghosh J.B., Roy M., Peters T. (2009). Thymoma associated with myasthenia gravis in infancy. Indian J. Pediatric.

[36] Bikhchandani J., Valusek P.A., Juang D., O’Brien J.E., Hetherington M., St Peter S.D. (2010). Giant ossifying malignant thymoma in a child. J. Pediatric Surg..

[37] Boylan E., Wyers M., Jaffar R. (2011). A rare case of thymoma in a 15-month-old girl. Pediatric Radiol..

[38] Rocha M.M., Neves P.D., Rodrigues C.C., Carrara G.F., Simões F.F., Etchebehere R.M., dos Santos J.P., Fatureto M.C. (2012). Invasive thymoma in a child: A rare case report. J. Pediatric Surg..

[39] Nikolic D.M., Nikolic A.V., Lavrnic D.V., Medjo B.P., Ivanovski P.I. (2012). Childhood-onset myasthenia gravis with thymoma. Pediatric Neurol..

[40] Iorio R., Evoli A., Lauriola L., Batocchi A.P. (2012). A B3 type-thymoma in a 7-year-old child with myasthenia gravis. J. Thorac. Oncol..

[41] Saha S., Suhani S., Basak A., Agarwal N., Dewan P. (2014). Pediatric thymoma with a difference: Report of a case and review of literature. J. Surg. Tech. Case Rep..

[42] Pacurar D., Tincu I., Muntean A., Lesanu G., Oraseanu D., Cordos I. (2016). Chest pain due to a giant thymoma in an adolescent boy. Acta Endocrinol. (Buchar.).

[43] Toret E., Demirag B., Köker S.A., Doyurgan O., Ergin M., Genc S., Karapinar T.H., Ay Y., Oymak Y., Vergin C. (2018). Aplastic anemia as an immune-mediated complication of thymoma: A case report. J. Pediatric Hematol. Oncol..

[44] Muthialu N., McHugh K., Slater O. (2018). Anterior mediastinal mass in a child-known but rare entity. Indian J. Surg..

[45] Figlewicz M.R., Bridwell R.E., Beal H., Cibrario A., Belcher C.N. (2020). Cardiomegaly masquerading as a pediatric thymoma: A case report. Cureus.

[46] Spigland N., Di Lorenzo M., Youssef S., Russo P., Brandt M. (1990). Malignant thymoma in children: A 20-year review. J. Pediatric Surg..

[47] Ramon y Cajal S., Suster S. (1991). Primary thymic epithelial neoplasms in children. Am. J. Surg. Pathol..

[48] Pescarmona E., Giardini R., Brisigotti M., Callea F., Pisacane A., Baroni C.D. (1992). Thymoma in childhood: A clinicopathological study of five cases. Histopathology.

[49] Carretto E., Inserra A., Ferrari A., Conte M., Di Cataldo A., Migliorati R., Cecchetto G., Bisogno G. (2011). Epithelial thymic tumours in paediatric age: A report from the TREP project. Orphanet J. Rare Dis..

[50] Yalçin B., Demir H.A., Ciftçi A.O., Orhan D., Varan A., Akyüz C., Kutluk T., Büyükpamukçu M. (2012). Thymomas in childhood: 11 cases from a single institution. J. Pediatric Hematol. Oncol..

[51] Rod J., Orbach D., Verité C., Coze C., Stephan J.L., Varlet F., Thomas-de-Montpreville V., Reguerre Y., Besse B., Sarnacki S. (2014). Surgical management of thymic epithelial tumors in children: Lessons from the French Society of Pediatric Oncology and review of the literature. Pediatric Blood Cancer.

[52] Stachowicz-Stencel T., Orbach D., Brecht I., Schneider D., Bien E., Synakiewicz A., Rod J., Ferrari A., Cecchetto G., Bisogno G. (2015). Thymoma and thymic carcinoma in children and adolescents: A report from the European Cooperative Study Group for Pediatric Rare Tumors (EXPeRT). Eur. J. Cancer.

[53] Liang X., Lovell M.A., Capocelli K.E., Albano E.A., Birch S., Keating A.K., Graham D.K. (2010). Thymoma in children: Report of 2 cases and review of the literature. Pediatric Dev. Pathol..

[54] Weissferdt A., Kalhor N., Bishop J.A., Jiang S.J., Ro J., Petersson F., Wu B., Langman G., Bancroft H., Bi Y. (2018). Thymoma: A clinicopathological correlation of 1470 cases. Hum. Pathol..

[55] Moran C.A., Weissferdt A., Kalhor N., Solis L.M., Behrens C., Wistuba I.I., Suster S. (2012). Thymomas I: A clinicopathological correlation of 250 cases with emphasis on the World Health Organization Schema. Am. J. Clin. Pathol..

[56] Weis C.A., Yao X., Deng Y., Detterbeck F.C., Marino M., Nicholson A.G., Huang J., Ströbel P., Antonicelli A., Marx A. (2015). The impact of thymoma histotype on prognosis in a worldwide database. J. Thorac. Oncol..

[57] Detterbeck F.C., Moran C., Huang J., Suster S., Walsh G., Kaiser L., Wick M. (2011). Which way is up? Policies and procedures for surgeons and pathologists regarding resection specimens of thymic malignancy. J. Thorac. Oncol..

[58] Bhora F.Y., Chen D.J., Detterbeck F.C., Asamura H., Falkson C., Filosso P.L., Giaccone G., Huang J., Kim J., Kondo K. (2014). The ITMIG/IASLC thymic epithelial tumors staging project: A proposed lymph node map for thymic epithelial tumors in the forthcoming 8th edition of the TNM classification of malignant tumors. J. Thorac. Oncol..

[59] Detterbeck F.C., Stratton K., Giroux D., Asamura H., Crowley J., Falkson C., Filosso P.L., Frazier A.A., Giaccone G., Huang J. (2014). The IASLC/ITMIG thymic epithelial tumors staging project: Proposal for an evidence-based stage classification system for the forthcoming (8th) edition of the TNM classification of malignant tumors. J. Thorac. Oncol..

[60] Nicholson A.G., Detterbeck F.C., Marino M., Kim J., Stratton K., Giroux D., Asamura H., Crowley J., Falkson C., Filosso P.L. (2014). The IASLC/ITMIG thymic epithelial tumors staging project: Proposals for the T component for the forthcoming (8th) edition of the TNM classification of malignant tumors. J. Thorac. Oncol..

[61] Kondo K., Van Schil P., Detterbeck F.C., Okumura M., Stratton K., Giroux D., Asamura H., Crowley J., Falkson C., Filosso P.L. (2014). The IASLC/ITMIG thymic epithelial tumors staging project: Proposals for the N and M components for the forthcoming (8th) edition of the TNM classification of malignant tumors. J. Thorac. Oncol..

[62] Moran C.A., Walsh G., Suster S., Kaiser L. (2012). Thymomas II: A clinicopathologic correlation of 250 cases with a proposed staging system with emphasis on pathologic assessment. Am. J. Clin. Pathol..

[63] Moran C.A. (2021). Thymoma Staging: An analysis of the different schemas. Adv. Anat. Pathol..

[64] Ruffini E., Fang W., Guerrera F., Huang J., Okumura M., Kim D.K., Girard N., Billè A., Boubia S., Cangir A.K. (2020). The international association for the study of lung cancer thymic tumors staging project: The impact of the eighth edition of the union for international cancer control and american joint committee on cancer TNM stage classification of thymic tumors. J. Thorac. Oncol..

[65] Liang G., Gu Z., Li Y., Fu J., Shen Y., Wei Y., Tan L., Zhang P., Han Y., Chen C. (2016). Comparison of the Masaoka-Koga staging and the International Association for the Study of Lung Cancer/the International Thymic Malignancies Interest Group proposal for the TNM staging systems based on the Chinese Alliance for Research in Thymomas retrospective database. J. Thorac. Dis..

[66] Imbimbo M., Ottaviano M., Vitali M., Fabbri A., Leuzzi G., Fiore M., Franceschini D., Pasello G., Perrino M., Schiavon M. (2018). Best practices for the management of thymic epithelial tumors: A position paper by the Italian collaborative group for ThYmic MalignanciEs (TYME). Cancer Treat. Rev..

[67] Kondo K., Monden Y. (2003). Therapy for thymic epithelial tumors: A clinical study of 1,320 patients from Japan. Ann. Thorac. Surg..

[68] Detterbeck F., Youssef S., Ruffini E., Okumura M. (2011). A review of prognostic factors in thymic malignancies. J. Thorac. Oncol..

[69] Ruffini E., Detterbeck F., Van Raemdonck D., Rocco G., Thomas P., Weder W., Brunelli A., Evangelista A., Venuta F., European Association of Thoracic Surgeons (ESTS) Thymic Working Group (2014). Tumours of the thymus: A cohort study of prognostic factors from the European Society of Thoracic Surgeons database. Eur. J. Cardiothorac. Surg..

