# Supplementary material for: Pediatric Thymoma: A Review and Update of the Literature
Source: Diagnostics (Basel). 2022 Sep 12;12(9):2205. doi: 10.3390/diagnostics12092205 (PMC9497562; doi:10.3390/diagnostics12092205)
Supplement: Supplementary file 1 [file diagnostics-12-02205-s001.zip › diagnostics-1829106-supplementary.pdf]

Table S1. Summary of cases.

Case	Reference	Age	Sex	Clinical Presentation	Thymoma Subtype	Masaoka–Koga Stage	UICC/AJCC TNM Classification (Stage)	Local Invasion	LN Involvement	Distant Metastasis	Treatment (Type of Resection)	Outcome	FU (Mos)
1	Figlewicz et al. [45]	16	F	chest discomfort<br>orthopnea<br>dyspnea	WHO B3	IIb<br>or<br>III[a]	T1aN0M0 (I)<br>or<br>T2N0M0 (II)[a]	inseparable from pericardium only on CT	no	no	surgery (NA)	alive, NED	0.27
2	Muthialu et al. [44]	5	NA	respiratory symptoms	WHO B2	I[b]	T1aN0M0 (I)[b]	no	no	no	surgery (R0)	alive, NED	NA
3	Toret et al. [43]	14	M	fever<br>coughing<br>sore throat<br>aplastic anemia	WHO A	I	T1aN0M0 (I)	no	no	no	surgery (R0)	alive, NED	21
4	Pacurar et al. [42]	17	M	chest pain	WHO B1	I[c]	T1aN0M0 (I)[c]	no	no	no	surgery (R0)	alive, NED	NA
5	Stachowicz–Stencel et al. [52]	10	F	NA<br>no AI/PN disorders	WHO A	I	T1aN0M0 (I)	no	no	no	surgery (R0)	alive, NED	60
6		14	F	NA<br>no AI/PN disorders	WHO AB	IIb	T1aN0M0 (I)	yes, not specified	no	no	surgery (R0)	alive, NED	84
7		15	M	NA<br>no AI/PN disorders	WHO AB	I	T1aN0M0 (I)	no	no	no	surgery (R0)	alive, NED	62
8		4	F	NA<br>no AI/PN disorders	WHO B1	IIb	T1aN0M0 (I)	yes, not specified	no	no	CTX + surgery (R0) + RT	alive, NED	51
9		7	F	NA<br>no AI/PN disorders	WHO B1	IIb	T1aN0M0 (I)	yes, not specified	no	no	surgery (R1) + CTX	alive, NED	60
10		6	M	NA<br>no AI/PN disorders	WHO B1	I	T1aN0M0 (I)	no	no	no	surgery (R0)	alive, NED	48
11		11	M	NA<br>no AI/PN disorders	WHO B1	I	T1aN0M0 (I)	no	no	no	surgery (R0)	alive, NED	12
12		16	F	MG<br>hypothoiridism	WHO B1	IIb	T1aN0M0 (I)	yes, not specified	no	no	surgery (R0)	alive, NED	48
13		7	M	NA<br>no AI/PN disorders	WHO B1	I	T1aN0M0 (I)	no	no	no	CTX + surgery (R0)	alive, NED	60
14		17	M	NA<br>no AI/PN disorders	WHO B1	I	T1aN0M0 (I)	no	no	no	surgery (R0)	alive, NED	72
15		5	F	NA<br>no AI/PN disorders	WHO B1	I	T1aN0M0 (I)	no	no	no	surgery (R0)	alive, NED	108
16		12	F	NA<br>no AI/PN disorders	WHO B1	IVb	TXNXM1b (IVb)	NA	NA	kidneys	autopsy	DOD	0.23
17		11	F	NA<br>no AI/PN disorders	WHO B1	I	T1aN0M0 (I)	no	no	no	surgery (R0)	alive, NED	60
18		13	M	NA<br>no AI/PN disorders	WHO B2	IIb	T1aN0M0 (I)	yes, not specified	no	no	surgery (R1 then R0)	alive, NED	60
19		15	F	NA<br>no AI/PN disorders	WHO B2	IIb	T1aN0M0 (I)	yes, not specified	no	no	surgery (R0)	alive, NED	132
20		13	M	MG	WHO B2	IVb	TXNXM1b (IVb)	NA	NA	peritoneum	unresectable: biopsy + CTX + surgery (R2)	DOD	13

21	Rod et al. [51]	15	F	asymptomatic	WHO AB	IIa	T1aN0M0 (I)	microscopic transcapsular invasion	no	no	surgery (R1)	alive, NED	48
22		7.5	M	respiratory symptoms	WHO B1	I	T1aN0M0 (I)	no	no	no	CTX + surgery (R0) + CTX	alive, NED	30
23		16	F	MG	WHO B1	IIb	T1aN0M0 (I)	yes, not specified	no	no	surgery (R0)	alive, NED	24
24		17	M	MG	WHO B1	I	T1aN0M0 (I)	no	no	no	surgery (R0)	alive, NED	36
25		13	M	respiratory symptoms	WHO B2	IIb	T1aN0M0 (I)	yes, not specified	no	no	surgery (R0)	alive, NED	12
26		12	M	vena cave syndrome	WHO B2	III	TXN0M0 (NA)	yes, not specified	no	no	unresectable: biopsy + CTX + surgery (R1) + CTX and RT	alive, NED	240
27	Saha et al. [41]	8	M	cough<br>chest pain	WHO B2	I	T1aN0M0 (I)	no	no	no	surgery (R0)	alive, NED	NA
28	Fonseca et al. [11]	16	M	cough<br>chest pain	WHO A	I	T1aN0M0 (I)	no	no	no	surgery (R0)	alive, NED	1
29		14	F	asymptomatic	WHO B1	I	T1aN0M0 (I)	no	no	no	surgery (R0)	alive, NED	1
30	Iorio et al. [40]	7	M	MG	WHO B3	IIa<br>or<br>IIb[d]	T1aN0M0 (I)	perithymic fat, not specified if macro– or microscopic invasion	no	no	surgery (R0)	alive, NED	72
31	Nikolic et al. [39]	14	M	MG	thymoma rich in lymphocytes	NA[e]	TXN0M0 (NA)[e]	no	no	no	surgery (R0)	alive, NED	24
32	Rocha et al. [38]	9	M	recurrent pneumonia	WHO B3	IVb[f]	T3N2M0 (IVb)[f]	mediastinal pleura, lung	1 peribronchial lymph node, 10th level	no	surgery (NA)	alive, NED	24
33	Yalçin et al. [50]	5	M	symptomatic<br>no AI/PN disorders	benign	NA[g]	TXN0M0 (NA)[g]	NA	no	no	surgery (NA)	alive, NED	166
34		12.5	M	symptomatic<br>ITP<br>SLE	benign	NA[g]	TXN0M0 (NA)[g]	NA	no	no	surgery (NA)	alive, NED	169
35		5	M	symptomatic<br>no AI/PN disorders	benign	NA[g]	TXN0M0 (NA)[g]	NA	no	no	surgery (NA)	alive, NED	163
36		11	F	symptomatic<br>no AI/PN disorders	benign	NA[g]	TXN0M0 (NA)[g]	NA	no	no	surgery (NA)	alive, NED	253
37		8	F	symptomatic<br>no AI/PN disorders	benign	NA[g]	TXN0M0 (NA)[g]	NA	no	no	surgery (NA)	alive, NED	367
38		5	M	symptomatic<br>no AI/PN disorders	benign	NA[g]	TXN0M0 (NA)[g]	NA	no	no	surgery (NA)	alive, NED	279
39		4	M	symptomatic<br>facial paralysis	invasive	NA[g]	TXN0M0 (NA)[g]	NA	no	no	surgery (NA) + CTX and RT	DOD (cervical LN metastases)	8
40		13	M	symptomatic<br>hypogammaglobulinemia	invasive	NA[g]	TXN0M0 (NA)[g]	NA	no	no	CTX and RT	alive, NED	40

41		13	M	symptomatic<br>no AI/PN disorders	invasive	NA[g]	TXN0M0 (NA)[g]	NA	no	no	CTX and RT + surgery (NA)	alive, NED	217
42		12	M	symptomatic<br>no AI/PN disorders	invasive	IVb[g]	TXN0M1b (IVb)[g]	NA	no	lung	CTX and RT + surgery (NA)	alive, NED	209
43		1	M	symptomatic<br>hyper IgE syndrome	invasive	NA[g]	TXN0M0 (NA)[g]	NA	no	no	none	DOD	0.8
44	Boylan et al. [37]	1.25	F	grunting episodes	WHO B2	I[c]	T1aN0M0 (I)[c]	no	no	no	surgery (R0)	alive, NED	8
45	Carretto et al. [49]	4.8	NA	cough	WHO B1	I	T1aN0M0 (I)	no	no	no	surgery (R0)	alive, NED	35.2
46		12.4	NA	dyspnea<br>coma	WHO B1	IVb	TXNXM1b (IVb)	NA	NA	kidneys	autopsy	DOD	0
47		11.4	NA	asymptomatic	WHO B1	I	T1aN0M0 (I)	no	no	no	surgery (R0)	alive, NED	93.4
48		15	NA	chest pain<br>fever	WHO AB	I	T1aN0M0 (I)	no	no	no	surgery (R0)	alive, NED	21.7
49	Liang et al. [53]	8	M	upper back pain	WHO B2	III<br>or<br>IVa<br>or<br>IVb[h]	T3N0M0 (IIIa)<br>or<br>T3N0M1a (IVa)<br>or<br>T2N0M1b (IVb)[h]	pericardium, diaphragm, and lingual of the left upper lobe of the lung only on CT	no	pericardium, diaphragm, lingual of the left upper lobe of the lung only on CT	CTX + surgery (R1) + CTX	32 months after treatment recurrent thymoma surgically resected (R0) + RT; alive, NED	56
50		15	F	cough<br>chest pain	WHO B1	I	T1aN0M0 (I)	no	no	no	surgery (R0)	alive, 12 months after treatment recurrent thymoma surgically resected (R1) + RT	12
51	Bikhchandani et al. [36]	10	F	difficult ventilation during operative procedure (excisional biopsy for bony lesion of the left tibia)	WHO B1	IVb[i]	TXN0M1b (IVb)i	NA	no	bone	unresectable: biopsy + CTX + surgery (R1)	alive, bony lesions unchanged	6
52	Ghosh et al. [35]	0.9	M	MG	WHO B2	III[j]	T3N0M0 (IIIa)<br>or<br>T4N0M0 (IIIb)[j]	great vessels	no	no	surgery (R2)	alive, evidence of disease but parents refused consent to CTX/RT	2
53	Coulter et al. [34]	6	M	cough<br>MG panel<br>presumptive diagnosis of congenital Isaacs syndrome	WHO B2	NA[k]	TXN0M0 (NA)[k]	adherent to the right pleural space, noninvasive and focally extending to the inked surgical margin	no	no	surgery (R1)	NA	NA
54	Honda et al. [33]	13	F	cough	WHO AB	I[l]	T1aN0M0 (I)[l]	no	no	no	surgery (R0)	alive, NED	8

				fever									
55	Rothstein et al. [32]	14	F	syncope	WHO AB	I	T1aN0M0 (I)	no	no	no	surgery (R1 then R0)	alive, NED	8
56	Trobaugh–Lotrario et al. [31]	16	M	left rib shoulder pain occasional “blue lips” tumor lysis syndrome	WHO B2	IVa[m]	T3N0M1a (IVa)[m]	separate tumor masses in the left paraspinal region (T6 and T7), along the left lateral chest wall and the surface of the diaphragm	no	multiple pleural implants	unresectable: biopsy + CTX + surgery (R2) + RT	alive, 1 month after treatment additional resection and RT for recurrent pleural disease. At 22 months from diagnosis, resection and CTX for pleural and peritoneal recurrence	22
57	Dhall et al. [30]	6	M	chest pain difficulty breathing nevus sebaceus	clusters of epithelial cells with admixed lymphocytes, a large cyst with epithelial lining, and Hassall’s corpuscles some of which cystic	I	T1aN0M0 (I)	no	no	no	surgery (R0)	alive, NED	36
58		13	F	asymptomatic constitutional t(6; 12) mental retardation biliary atresia	extensive necrosis, cystic degeneration, and epithelial tumor cells embedded in collagenous stroma enclosed by an intact fibrous capsule; aggregates of lymphoid tissue and remnants of normal thymic tissue at the periphery	I	T1aN0M0 (I)	no	no	no	surgery (R0)	alive, NED	36
59	Sicherer et al. [29]	15	F	oral candidiasis proximal muscle weakness fever respiratory distress cellular immunodeficiency MG	necrotic, hyalinized, and calcified mass with small peripheral regions of classic lymphocyte–predominant and spindle cell thymoma	I[c]	T1aN0M0 (I)[c]	no	no	no	surgery (R0)	alive, NED	12
60	Lam et al. [28]	9	M	chronic cough	invasive	NA[k]	TXN0M0 (NA)[k]	NA	no	no	surgery (NA)	alive, NED	NA
61		11	F	asymptomatic	thymoma of cortical predominance	NA[k]	TXN0M0 (NA)[k]	partly encapsulated with capsular invasion	NA	no	surgery (NA)	alive, NED	NA

62	Kaplinsky et al. [27]	8	M	vena cave syndrome	lobulated tumor with clusters of large, ovoid epithelial cells and small amounts of plasma cells and lymphocytes	III[n]	T3N0M0 (IIIa)[n]	upper lobe of the right lung, pericardium, superior vena cava	no	no	surgery (R2) + RT and CTX	DOD (left pulmonary and skeletal metastases)	6
63		13	F	vena cave syndrome	clusters of large, ovoid epithelial cells and small amounts of plasma cells and lymphocytes	III[n]	T4N0M0 (IIIb)[n]	extended posteriorly on both sides of the trachea with pressure on the superior vena cava	no	no	unresectable: biopsy + CTX and RT	alive, NED	84
64	Pescarmona et al. [48]	15	M	NA<br>no MG	lymphocyte–rich cortex–like tissue with no obvious medullary differentiation classified as cortical thymoma	I<br>or<br>IIa[o]	T1aN0M0 (I)	encapsulated with microscopic capsular infiltration	no	no	surgery (R0)	alive, NED	72
65		11	F	NA<br>no MG	lobulated tumor with lymphocyte–poor areas of medullary differentiation, prominent Hassall's corpuscles, and lymphocyte–rich cortex–like tissue	I	T1aN0M0 (I)	no	no	no	surgery (R0)	alive, NED	108
66		12	F	NA<br>no MG	lobulated tumor with lymphocyte–poor areas of medullary differentiation, prominent Hassall's corpuscles, and lymphocyte–rich cortex–like tissue	I<br>or<br>IIa[o]	T1aN0M0 (I)	encapsulated with microscopic capsular infiltration	no	no	surgery (R0)	alive, NED	84
67		13	M	NA<br>no MG	lobulated tumor with lymphocyte–poor areas of medullary differentiation, prominent Hassall's corpuscles, and lymphocyte–rich cortex–like tissue	I	T1aN0M0 (I)	no	no	no	surgery (R0)	alive, NED	12

68		15	M	NA<br>no MG	lobulated tumor with lymphocyte–poor areas of medullary differentiation, prominent Hassall's corpuscles, and lymphocyte–rich cortex–like tissue	I	T1aN0M0 (I)	no	no	no	surgery (R0)	alive, NED	3
69	Ramon y Cajal et al. [47]	14	F	asymptomatic	lymphocyte–rich thymoma with biphasic cellular composition, perivascular spaces, areas of medullary differentiation, and focal microcystic areas	I	T1aN0M0 (I)	no	no	no	surgery (R0)	alive, NED	30
70		1	M	respiratory distress	lymphocyte–rich thymoma with unusual stromal features, organoid growth pattern, irregular expanded lobules, and interconnecting strands of epithelial cells; perivascular spaces with prominent fibrosis, hyalinization, and Hassall's corpuscles	I	T1aN0M0 (I)	no	no	no	surgery (R0)	NA	NA
71		14	M	chest pain<br>pleural effusion	lymphocyte–rich thymoma with unusual stromal features, organoid growth pattern, irregular expanded lobules, and interconnecting strands of epithelial cells; secondary cystic changes; perivascular spaces with prominent fibrosis and hyalinization	I	T1aN0M0 (I)	no	no	no	surgery (R0) + RT and CTX	NA	NA

72		12	F	respiratory distress hypoplastic anemia	lymphocyte–rich thymoma with unusual stromal features, organoid growth pattern, irregular expanded lobules, and interconnecting strands of epithelial cells; abundant plasma cells and hyperplastic lymphoid follicles; secondary cystic changes and Hassall's corpuscles	I	T1aN0M0 (I)	no	no	no	surgery (R0)	alive, NED	8
73		16	F	respiratory distress vena cave syndrome	lymphocyte–rich thymoma with unusual stromal features, organoid growth pattern, irregular expanded lobules, and interconnecting strands of epithelial cells; perivascular spaces and Hassall's corpuscles	NA[e]	TXN0M0 (NA)[e]	NA	no	no	surgery (R2) + RT and CTX	alive, NED	36
74		9	M	shortness of breath	atypical spindle cell thymoma with spindle cells, scant lymphocytes, and areas with hemangiopericytoma–like appearance; the pulmonary metastasis showed the features of conventional lymphocytes rich thymoma	IVb	TXNXM1b (IVb)	NA	NA	bone, lung	surgery (R2) + RT and CTX	alive, NED	72
75		8	M	chest pain	atypical spindle cell thymoma with a dense population of oval to spindle cells, often separated by large cavernous vascular spaces, areas with hemangiopericytoma–	IVb	TXNXM1b (IVb)	infiltration of adjacent structures with compression of vessells and nerves	NA	lung	surgery (R2) + CTX	alive, local recurrence after 15 months	15

					like appearance, and small areas of necrosis								
76	Aghaji et al. [25]	3.6	M	cough<br>wheezing<br>shortness of breath<br>MG	lymphoepithelial thymoma	III[p]	T4N0M0 (IIIb)[t]	all the superior mediastinal viscera, innominate vein, great vessels, phrenic nerves, trachea, bronchi	no	no	surgery (R2)	DOD	0.8
77	Spigland et al. [46]	15	F	vena cave syndrome	lymphoepithelial thymoma	III	T3N0M0 (IIIa)	pleura, pericardium, innominate vein, superior vena cava	no	no	steroids and RT unresectable: biopsy + RT and CTX	DOD (liver and bone metastases)	30
78		3	M	cough<br>dyspnea<br>wheezing	lymphocytic thymoma	III	T3N0M0 (IIIa)	pericardium, pleura, lung, innominate vein, superior vena cava	no	no	steroids and RT unresctable: surgery (R2) + RT and CTX	DOD (soft tissue metastases and spinal canal infiltration)	18
79		9	M	vena cave syndrome	thymoma with epithelial predominance	III	T2N0M0 (II)	pleura, pericardium	no	no	unresectable: biopsy + RT and CTX	DOD (liver, bone, and mediastinal metastases)	7
80	Watts et al. [26]	8.25	M	cough<br>chest pain<br>wheezing<br>hypogammaglobulinemia<br>lymphopenia	sheets of oval epithelial cells, small aggregates of mature lymphocytes, and perivascular spaces occupied by plasma cells and few lymphocytes	I[c]	T1aN0M0 (I)[c]	no	no	no	surgery (R0)	died of disseminated varicella infection	4
81	Shibata et al. [24]	6	M	asymptomatic	lobulated tumor with a mixture of epithelial cells and lymphocytes, marked infiltration of eosinophils, necrosis, scattered mono–multinucleated large cells and mitotic figures of tumor cells; calcification,	III[q]	T3N0M0 (IIIa)[q]	pericardium, left upper lobe of the lung, innominate vein	no	no	surgery (R2) + RT	alive, NED	10

					microcystic degeneration, and Hassal's corpuscles								
82	Furman et al. [23]	4.8	F	respiratory symptoms MG	diffuse mixture of morphologically normal small lymphocytes and much larger epithelial cells; microcystic degeneration, perivascular clear spaces, and foci of larger cysts containing proteinaceous material	I[c]	T1aN0M0 (I)[c]	no	no	no	surgery (R0)	alive, NED	12

AI/PN, autoimmune and paraneoplastic associated disorders; CT, computed tomography; CTX, chemotherapy; DOD, died of disease; F, female; FU, follow–up; ITP, idiopathic thrombocytopenic purpura; LN, lymph node; M, male; MG, myasthenia gravis; Mos, months; NA, not available; NED, not evidence of disease; R0, complete resection; R1, microscopically incomplete resection; R2, macroscopically incomplete resection; RT, radiotherapy; SLE, systemic lupus erythematosus; UICC/AJCC TNM, Union for International Cancer Control/American Joint Committee on Cancer Tumor–Node–Metastasis; WHO, World Health Organization.

[a] Classified as Masaoka stage III by the authors, however diagnosis was made on needle biopsy and adherence to the pericardium was highlighted only with computed tomography.

[b] Classified as clinical stage IIa by the authors, however they reported a mass limited within the capsule.

[c] No staging available, however the authors described a non–invasive, capsulated mass.

[d] Classified as Masaoka stage II by the authors, however they reported perithymic fat invasion without specifying whether micro– or macroscopic.

[e] Classified as Masaoka stage II by the authors, however the description is unclear.

[f] Classified as Masaoka stage IVa by the authors, however one peribronchial lymph node (10th level) metastasis was present.

[g] Classified as benign or invasive by the authors; in Case 42 there was lung metastasis.

[h] Classified as Masaoka stage IVa by the authors, however they reported pericardium and lung involvement without other details (direct invasion or separate nodules?).

[i] Classified as Masaoka stage I by the authors, however they described a bony lesion.

[j] No staging available, however the authors reported an unspecified invasion of the great vessels.

[k] No staging available and unclear description.

[l] No staging available, however the authors described a well circumscribed cystic lesion.

[m] No staging available, however the authors reported multiple pleural implants and separate tumor masses along the chest wall.

[n] No staging available: in Case 62 there was a mass infiltrating the upper lobe of the right lung, the pericardial sac, and the superior vena cava; in Case 63, a mass infiltrating both sides of the trachea with pressure on the superior vena cava was present.

[o] Classified as Masaoka stage II by the the authors, however they reported foci of microscopic capsular infiltration without other details (infiltration into or through the capsule?).

[p] No staging available, however the authors reported invasion of all the superior mediastinal viscera, innominate vein, great vessels, phrenic nerves, trachea, and bronchi.

[q] No staging available, however the authors reported invasion of the pericardium, the left upper lobe of the lung, and the innominate vein.
